# Congruence and Diversity of Butterfly-Host Plant Associations at Higher Taxonomic Levels

**DOI:** 10.1371/journal.pone.0063570

**Published:** 2013-05-23

**Authors:** José R. Ferrer-Paris, Ada Sánchez-Mercado, Ángel L. Viloria, John Donaldson

**Affiliations:** 1 Kirstenbosch Research Centre, South African National Biodiversity Institute, Cape Town, Western Cape, Republic of South Africa; 2 Botany Department, University of Cape Town, Cape Town, Western Cape, Republic of South Africa; 3 Centro de Estudios Botánicos y Agroforestales, Instituto Venezolano de Investigaciones Científicas, Maracaibo, Estado Zulia, Venezuela; 4 Centro de Ecología, Instituto Venezolano de Investigaciones Científicas, Caracas, Distrito Capital, Venezuela; University Copenhagen, Denmark

## Abstract

We aggregated data on butterfly-host plant associations from existing sources in order to address the following questions: (1) is there a general correlation between host diversity and butterfly species richness?, (2) has the evolution of host plant use followed consistent patterns across butterfly lineages?, (3) what is the common ancestral host plant for all butterfly lineages? The compilation included 44,148 records from 5,152 butterfly species (28.6% of worldwide species of Papilionoidea) and 1,193 genera (66.3%). The overwhelming majority of butterflies use angiosperms as host plants. Fabales is used by most species (1,007 spp.) from all seven butterfly families and most subfamilies, Poales is the second most frequently used order, but is mostly restricted to two species-rich subfamilies: Hesperiinae (56.5% of all Hesperiidae), and Satyrinae (42.6% of all Nymphalidae). We found a significant and strong correlation between host plant diversity and butterfly species richness. A global test for congruence (Parafit test) was sensitive to uncertainty in the butterfly cladogram, and suggests a mixed system with congruent associations between Papilionidae and magnoliids, Hesperiidae and monocots, and the remaining subfamilies with the eudicots (fabids and malvids), but also numerous random associations. The congruent associations are also recovered as the most probable ancestral states in each node using maximum likelihood methods. The shift from basal groups to eudicots appears to be more likely than the other way around, with the only exception being a Satyrine-clade within the Nymphalidae that feed on monocots. Our analysis contributes to the visualization of the complex pattern of interactions at superfamily level and provides a context to discuss the timing of changes in host plant utilization that might have promoted diversification in some butterfly lineages.

## Introduction

Plant feeding insects make up a large part of the earths total biodiversity so that explaining mechanisms behind the diversification of these groups could promote the understanding of global biodiversity [1]. A seminal paper about coevolution between butterflies and host plants by Ehrlich and Raven [2] triggered intensive discussions about the role of biotic interactions in the evolutionary processes that led to radiation in species numbers.

There are two key predictions in Ehrlich and Raven’s coevolution scenario. The first is that related butterflies tend to feed on related host plants as a consequence of a stepwise coevolutionary process in which plants evolve defenses against herbivores and these herbivores, in turn, evolve new capacities to cope with the defenses. Insects that manage to colonize plants with novel defenses would enter a new adaptive zone and could in turn diversify onto the relatives of this plant, because they will be chemically similar. The second prediction is that there should be a general correlation between host diversity and herbivore species richness as a consequence of the adaptive radiation and enhanced diversification experienced by insect lineages due to the adaptation to diverse, chemically distinct plant clades [3].

Later on it was recognized that other evolutionary scenarios could also explain the patterns observed. Herbivores and plants can radiate in separate bursts following the evolution of novel defenses and counter-defenses (escape-radiate scenario), or follow a sequence of independent host diversification followed by colonization and radiation of herbivores (sequential evolution). Both scenarios might result in some degree of congruence between the cladograms of insects and their host plants, but strict congruence appears to be rare among insect herbivores [3], [4]. This is probably because plant diversification preceded herbivore radiation and insect plant recognition mechanisms might focus on phytochemical cues that are not necessarily related to host plant taxonomy [5], [6].

More recently, a broad-scale phylogenetic analysis of butterflies [7] found that host shifts were more common between closely related plants and that there is a higher tendency to recolonize ancestral hosts. These results led them to propose the oscillation hypothesis as an alternative mechanism to explain the patterns in host plant associations [8]. They argue that dynamic oscillations in host range, instead of a steady process of specialization and cospeciation, is the principal driver of the high diversity of plant feeding insects. However, the assumptions and predictions of the oscillation hypothesis have been tested in only one butterfly family [7], [9].

Besides the mechanism for diversification, the direction of evolution of host plant associations is profoundly dependent on the ancestral character [5]. Ehrlich and Raven [2] proposed a unique ancestral host plant for true butterflies (Papilionoidea, but excluding Hesperiidae and Hedylidae) and it was most likely a primitive angiosperm in the lineage of the Aristolochiaceae. Later revision of host plant associations from different regions suggested a common ancestral plant clade near the Malvaceae that would explain the range of host plants used by butterflies in the families Hedylidae, Hesperiidae and Nymphalidae, but not the associations of Pieridae and Papilionidae [10]. More recently, Janz and Nylin [7] proposed that the ancestral host plant of Papilionoidea appeared to be within a highly derived clade in the plant subclass Rosidae, including the family Fabaceae.

Tests to determine whether hypotheses about the evolution of insect-host plant associations and ancestral host plant are generally applicable, or even if they apply to the butterfly lineages from which support has previously been found, has been limited because of the scarcity of extensive datasets and comprehensive phylogenies [11]. The first general and global account of butterfly host plant associations outlined by Ehrlich and Raven [2] was purely qualitative. Some authors have provided quantitative or semi-quantitative analyses focused on describing taxonomic or regional patterns in host plant use for particular butterfly families or regions [12]–[14]. Semi-quantitative data in the form of binary association indices have been used in several phylogenetic analyses, sometimes removing uncommon observations [15]–[18]. Recent efforts to compile several data sources [19]–[21] and provide access to these compilations in on-line databases and other web-based resources, have improved the availability of the data [e.g. HOST, Caterpillar, and FUNET databases]. However, there have been few published quantitative analyses based on these sources [9], [22], [23], probably because this kind of dataset needs to be carefully revised and validated to avoid negative effects of biased or incomplete information [9], [14], [23].

In this paper we provide an updated quantitative summary of host plant associations for all butterfly families, based on updated and validated data from different sources. We focus on higher taxonomic levels (butterfly subfamilies and Angiosperm orders) in order to evaluate whether macro-evolutionary patterns of host plant associations can be detected in a large-scale analysis encompassing the phylogenetic relationships of all butterfly families [24]. Specifically, we want to evaluate: (1) is there a general correlation between host diversity and butterfly species richness? (2) whether evolution of host plant use has followed consistent patterns across butterfly lineages, and (3) what is the common ancestral host plant for each butterfly lineage?

## Methods

### Butterfly Phylogeny, Taxonomy and Host Plant Associations

Traditionally the clade “Rhopalocera” was considered as a monophyletic group within the Lepidoptera, comprising three distinct superfamilies: Papilionoidea (five families of “true butterflies”), Hesperioidea (“skippers”, one family) and Hedyloidea (“butterfly moths”, one family) [25]. Recent combined morphological and molecular analysis suggests that the “true butterflies” are paraphyletic and the superfamily Papilionoidea has been redefined to include all seven families [26], [27]. For simplicity we will refer to all seven families collectively as “butterflies”.

We compiled a tentative global checklist of butterfly species from different sources, including authoritative checklists that have been published or made available in electronic format by several authors (e.g. GloBIS/GART, http://www.globis.insects-online.de/species; The Lepidoptera Taxome Project, http://www.ucl.ac.uk/taxome/; Nymphalidae.net, http://www.nymphalidae.net/home.htm; Afrotopical butterflies, http://www.atbutterflies.com/index.htm) and published catalogues [28], [29]. For several taxonomic groups not yet included in such lists, we used information from the best available sources (Encyclopedia of life, EOL, http://www.eol.org; Lepidoptera Phylogeny, LepTree, http://www.leptree.net/; Tree of Life, http://tolweb.org/tree/; Lepidoptera and some other life forms at FUNET, ftp://www.nic.funet.fi/index/Tree_of_life/intro.html) and carefully checked to remove duplicates or inconsistent nomenclature. All species were assigned to one of five regions according to distributional information obtained from the previous sources and the Global Biodiversity Information Facility (http://www.gbif.org/). These broad regions reflect a very crude approximation to the major biogeographical division of butterflies [30]–[32] and were used here only as a reference of geographical zones where butterfly research can be summarized consistently: Oriental (OR), Nearctic (NC), Neotropical (NT), Afrotropical (AT) and Palearctic (PA). Species with their main distribution in one region and only marginally represented in another region were assigned to the main region. When it was not possible to determine a main region, or when the species was present in more than two regions, we classified it as “widespread” (W).

We used four types of sources to compile a list of butterfly-host plant associations. The first source was the *Lepidoptera Host Plant* database (http://www.nhm.ac.uk/hosts) that made a systematic compilation of information from literature references worldwide. The second source was *FUNET*, which also provides several summarized, well-documented, literature-based records at worldwide scale. The third source was a series of study-site databases that have been compiled from field rearing records of caterpillars and their host plants. These include the *Caterpillar Data Base* (http://caterpillars.unr.edu/) and the project *Inventory of the macrocaterpillar fauna and its food plants and parasitoids of Area de Conservación Guanacaste* (http://janzen.sas.upenn.edu) that together comprise information from Costa Rica, Ecuador, Brazil, and the United States. Finally, we digitalized host plant records from published sources for selected species and regions that were underrepresented in other sources [10], [31], [33]–[37].

The initial compilation comprised all records listed in the referenced sources, including angiosperm and non-angiosperm plants, detritus and animal food sources. We validated and updated plant names at species, genus or family level by using the taxonomic and nomenclatural information tools provided on the *Phylomatic* home page (http://www.phylodiversity.net/phylomatic/), The *Plant List* (http://www.theplantlist.org/), and additional information on the Angiosperm Phylogeny Website (http://www.mobot.org/MOBOT/Research/APweb/welcome.html). Taxonomic validation for butterfly names was based on the previously compiled checklist of butterfly species. This compilation includes records with different levels of taxonomic resolution for both the host plant (order, family, genus, species), and the butterfly (genera, species), but in this analysis we focus on higher-level relationships and thus summarize the information at the level of plant orders and butterfly subfamilies.

### Phylogenies

We used the updated phylogeny of angiosperm plant orders (APGIII) provided by The Angiosperm Phylogeny Group [38]. In this APGIII, the Aristolochiaceae of Ehrlich and Raven [2] is located in the order Piperales within the magnoliid clade, the Malvales of Ackery [10] and the rosid clade of Janz and Nylin [7] correspond loosely to the malvid and fabid clades within the rosids.

For butterflies, we combined information from higher level classification of families [25], [26] and lower level classification of subfamilies (from LepTree and TOL) to build three tentative cladograms that reflect the current views derived from traditional classifications (mostly based on adult and early stage morphology) [12], [25], and recent phylogenetic analyses based on a combination of morphological and molecular data [26], [39]–[42].

**Figure 1:**
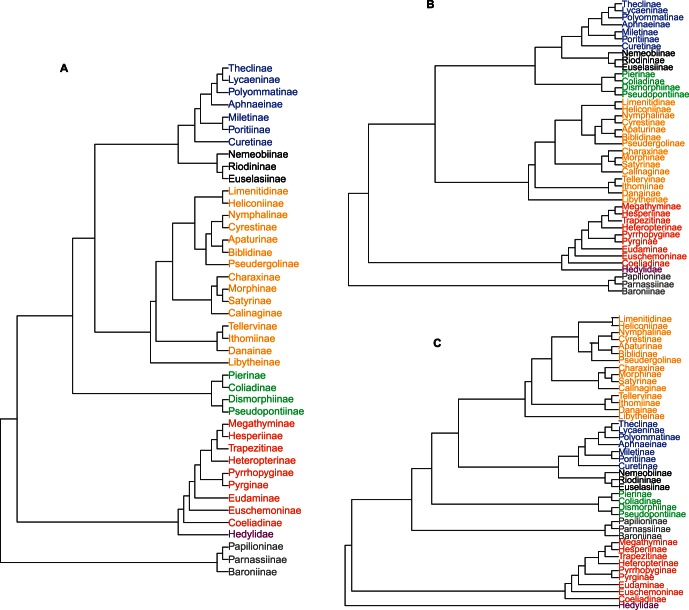
Three alternative phylogenetic relationships among butterfly families and subfamilies. Based on Heikkilä et al. [27] and Kristensen et al. [26]. **A**) Alternative 1 cladogram, **B**) alternative 2 cladogram, **C**) traditional cladogram.

The recent proposal to combine all seven families in a single superfamily [27] is based on the work of Heikkilä et al. [26], which proposes Papilionidae as a basal group to a clade formed by Hesperiidae (skippers) and Hedylidae (butterfly moths), and the four remaining families. Riodinidae and Lycaenidae have been confirmed as close but distinct sister groups, but the position of Pieridae is ambiguous, suggesting two alternative hypotheses: that Pieridae is the sister group to Lycaenidae+Riodinidae (“alternative 1” cladogram in Fig. 1A); or that Pieridae is the sister group to Nymphalidae+Lycaenidae+Riodinidae (“alternative 2” cladogram in Fig. 1B). For the sake of comparison, the traditional view of three separate superfamilies, with Papilionidae and Pieridae families as basal clades within the Papilionoidea [25], is represented as a “traditional” cladogram (Fig. 1C).

In the lower level classification we followed current views in most groups, except in some tribes with distinct host plant associations. Thus we retained the traditional Morphinae (Morphini and Brassolini tribes) as a sister clade of Satyrinae, and the subfamily status for Danainae, Ithominae and Tellervinae; we also retained the Pyrrhopyginae (Oxynetrini, Passovini, Pyrrhopygini and Zoniini tribes) as a sister group to Pyrginae, and Megathyminae as a distinct subfamily.

For all cladograms we computed branch lengths using the method of Grafen [43]. We provide a dataset ( Dataset S1) with the summaries of host plant associations per butterfly genus and subfamily and the final phylogenies of the plant orders and butterfly subfamilies used in the current analysis.

### Analysis

#### Representativeness and biases

We evaluated representativeness and biases of the compiled information by measuring three aspects: (1) proportion of butterfly species with host plant information across regions and butterfly families; (2) number of erroneous or discarded records including typing errors, non-resolved taxonomy, or records with general terms such as “grasses” or “palms”, or ambiguous references to orders (or other higher level classification terms) that might have changed in circumscription; and (3) number of plant families recorded, and the plant families, genera and species more frequently used.

#### Association matrices

For the analysis we built association matrices between plant orders (rows) and butterfly subfamilies (columns) and a single measure of association strength in each cell [44]. We use upper case bold letters to denote the association matrix and lower case italic letters to refer to the index of association strength.

For most analyses we consider two association matrices, either matrix **A** based on a binary association index *a_ij,_* which simply measures absence (0) or presence (1) of association, or matrix **C** based on a quantitative measure of association strength *c_ij_* representing the number of butterfly species from subfamily *j* feeding on host plant order *i*.

To compare the relative importance of host plant orders for each butterfly subfamily, we calculated a matrix of proportions **Z**, based on the index *z_ij_ = c_ij_/S_j_*, where *S_j_* is the number of butterfly species in subfamily *j* that have at least one host plant record in the compilation. It is important to note that since many species were polyphagous, and can use host plants from more than one order, the sum of *z_ij_* values for a particular subfamily does not necessarily add up to one. We consider that an order *i* was important for a subfamily *j* if *z_ij_>0.1*, and the term “most important resource” was used for the order with the highest value of *z_ij_* for a particular subfamily *j*. Cases where an order was used by most species in a butterfly subfamily (*z_ij_>0.9*) were further recognized and are referred to as a “primary resource” even if many species in that subfamily might use additional orders as well.

For some analyses we used matrix **X**, based on a binary index *x_ij_* that represents only the “important” associations between host plant orders and butterfly subfamilies, and is equal to 1 if *z_ij_>0.1* and 0 otherwise.

#### Host plant diversity and species richness

We estimated host plant diversity by three different methods. First we estimated the total number of host plant species (*h* = sum of columns in association matrix **A**) used by all the members of each butterfly subfamily. Second, we fitted a Fisher’s log-series to the columns of the association matrix **C** and estimated the value of the parameter α [14]. These measures do not take the phylogenies of plant orders into account. Third, we calculated a Faith’s index of Phylogenetic Diversity (PD) based on the binary association matrix **A** and the branch lengths of the phylogeny for plant orders [45]. We compared the calculated value of PD with the expected PD value of a sample of plant orders of equivalent size drawn at random from the plant phylogeny [46].

We calculated Pearson’s product moment correlation between each measure of host plant diversity with the logarithm of species richness for each butterfly subfamily (*R_j_* as defined above), using phylogenetically independent contrasts calculated from the butterfly cladograms and scaled with their expected variance [47].

#### Congruence in phylogenies

We used the ParaFit test to measure the congruency between host plant and butterfly phylogenies [48]. Congruence refers to the degree to which the herbivores and their hosts occupy corresponding positions in the phylogenetic trees. The test is based on a binary association matrix and contrasts the observed pattern against the null hypothesis of independent evolution (ParaFitGlobal).

We used a jackknife method to test the significance of individual links against the null hypothesis of random association (ParaFitLink2). We applied the test to the unweighted and weighted binary interaction matrices (**A** and **X**).

#### Ancestral character estimation

We grouped butterfly subfamilies according to the main patterns in host plant use and we estimated the ancestral character state using a maximum likelihood method [49]. We assigned each butterfly subfamily to the resource used by most species: non-angiosperms, magnoliids, monocots, basal eudicots, and core-eudicots (fabids, malvids, and asterids), and animal (entomophagous). We consider that non-angiosperm hosts and animal resources are derived states [2; but see 50], with transition rates in one direction from angiosperm to the derived states, but the transition rates among angiosperms might be variable [7]. We considered three models to tests this hypothesis: the null model with constant transition rates among angiosperm groups (*one single rate*); a full model with different transition rates within basal groups (magnoliids, monocots and basal eudicots), from basal groups to core-eudicots, and from core-eudicots to basal groups (*three rates*); and a simplified model where the transition rates from core-eudicots to the basal groups and within basal groups are constant, but the transition rates from basal groups to core-eudicots are different (*two rates*). We used Akaike Information Criterion (AIC) to compare models [51].

All the statistical analyses were performed with the free statistical software R [http://cran.r-project.org/, version 2.5.14], and Phylocom [52], and R-packages *picante*, *ape* and *vegan* [52]–[54].

## Results

### Representativeness and Biases of the Database

**Figure 2:**
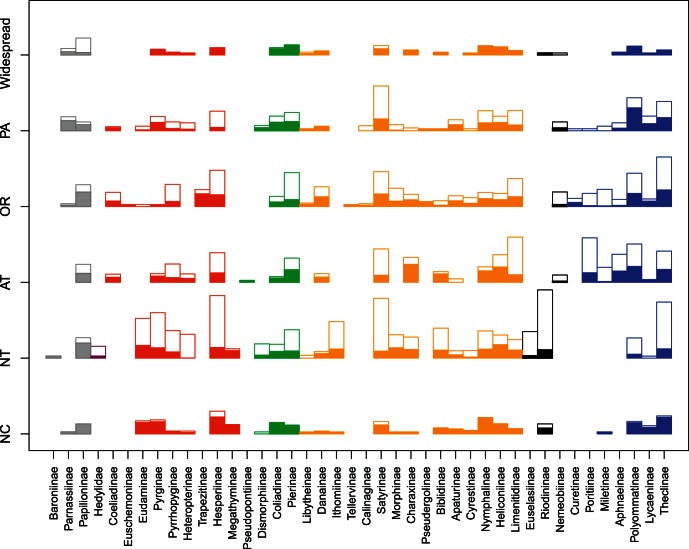
Geographical and taxonomical representativeness of host plant association data. Block height is proportional to the square root of the number of butterfly species among regions and subfamilies. Solid blocks represent the number of species with host plant records. Open blocks represent the number of species without host plant records. Grey: Papilionidae. Dark red: Heylidae. Red: Hesperiidae. Green: Pieridae. Orange: Nymphalidae. Blue: Lycaenidae. Black: Riodinidae.

The global checklist compiled for this work includes 17,854 species from 1,804 genera (Table 1). Except for the Hedylidae, all butterfly families were represented worldwide, but with regional differences in species richness (Fig. 2). The Nymphalidae was the largest of all butterfly families with 5,921 species worldwide (5,339 with distribution information), but better represented in NT (40.3% of the species) and AT (23.4%). Most subfamilies were present in NT, but Satyrinae, Ithominae and Biblidinae were the most important. In contrast, only eight subfamilies were represented in AT, with Limenitidinae, Satyrinae, Heliconiinae and Charaxinae being the most important. The subfamilies with the most restricted distribution within Nymphalidae were Tellervinae, with one species in OR, and Calinaginae with eight species between OR and PA.

**Table 1:** Taxonomic representation of butterflies in the compilation.

		Number of genera			Number of species		
Family	Subfamily	World wide*	Compilation	Proportion	World wide*	Compilation	Proportion
Hedylidae		1	1	1.000	36	6	0.167
Hesperiidae	Coeliadinae	8	6	0.750	89	33	0.371
	Eudaminae	50	43	0.860	430	159	0.370
	Euschemoninae	1	1	1.000	1	1	1.000
	Hesperiinae	314	188	0.599	2,020	462	0.229
	Heteropterinae	11	6	0.545	182	15	0.082
	Megathyminae	5	5	1.000	39	36	0.923
	Pyrginae	86	62	0.721	642	209	0.326
	Pyrrhopyginae	67	37	0.552	490	100	0.204
	Trapezitinae	18	14	0.778	75	52	0.693
Papilionidae	Baroniinae	1	1	1.000	1	1	1.000
	Papilioninae	20	20	1.000	400	237	0.593
	Parnassiinae	8	7	0.875	61	43	0.705
Pieridae	Coliadinae	18	15	0.833	180	112	0.622
	Dismorphiinae	7	5	0.714	58	14	0.241
	Pierinae	59	46	0.780	761	258	0.339
	Pseudopontiinae	1	1	1.000	1	1	1.000
Lycaenidae	Aphnaeinae	17	13	0.765	286	92	0.322
	Curetinae	1	1	1.000	18	8	0.444
	Lycaeninae	6	4	0.667	110	60	0.545
	Miletinae	13	12	0.923	188	40	0.213
	Polyommatinae	121	93	0.769	1,477	523	0.354
	Poritiinae	56	35	0.625	721	109	0.151
	Theclinae	216	137	0.634	2,276	607	0.267
Riodinidae	Euselasiinae	5	2	0.400	171	16	0.094
	Nemeobiinae	13	6	0.462	82	15	0.183
	Riodininae	122	51	0.418	1,138	155	0.136
Nymphalidae	Apaturinae	19	16	0.842	87	43	0.494
	Biblidinae	39	27	0.692	275	95	0.345
	Calinaginae	1	1	1.000	10	1	0.100
	Charaxinae	20	17	0.850	342	180	0.526
	Cyrestinae	3	3	1.000	46	13	0.283
	Danainae	12	9	0.750	167	76	0.455
	Heliconiinae	43	37	0.860	562	275	0.489
	Ithomiinae	43	29	0.674	339	81	0.239
	Libytheinae	2	2	1.000	10	5	0.500
	Limenitidinae	48	37	0.771	1,023	232	0.227
	Morphinae	36	25	0.694	245	84	0.343
	Nymphalinae	55	47	0.855	509	254	0.499
	Pseudergolinae	4	4	1.000	7	7	1.000
	Satyrinae	233	126	0.541	2,292	441	0.192
	Tellervinae	1	1	1.000	7	1	0.143
Totals		1,804	1,193	0.661	17,854	5,152	0.289

Lycaenidae was the second largest butterfly family, with 5,076 species (4,109 with distribution information), most of them present in AT (33.7%), and OR (26.1%) regions. All subfamilies were present in AT except Curetinae, and most species were in the Poritinae, Theclinae and Polyommatinae subfamilies, while in OR and NT Theclinae were clearly dominant.

Hesperiidae was a medium-sized family (3,968 species, 3,562 with distribution information) with a large proportion in NT (61.7%). Within NT, Hesperiidae and Pyrginae were richer in species, but Pyrrhophyginae, Heteropteriinae and Eudaminae were also well represented. In all other regions the Hesperiinae was the most important subfamily, while the Trapezitinae, Euchemoninae and Coeliadinae were mainly distributed in, or restricted to, the OR region.

Riodiniidae (1,391 species, 1,381 with distribution information) was mostly restricted to a single region, with up to 92.2% of the species in NT, and only 107 species in the other regions, including 51 in OR region.

The majority of Pieridae (1,000 species, 984 with distribution information) were distributed in OR (30.2%) and NT (28.8%), with most species in the subfamily Pierinae. Papilionidae (462 species, 444 with distribution information) were also mainly distributed in OR (25.2%) and NT (21.8%), but they also had an important number of widespread species (17%), with Papilioninae being the most important subfamiliy. Hedylidae was barely represented by 36 species restricted to the NT region (Fig. 2).

The Neotropical region had a high number of species with host plant records (1,500), but they represent only 40.9% of the fauna of the region. On the other hand, NC had the highest proportion of species with host plant records (92%). Among butterfly families, Papilionidae was the best represented with 59% of the species with information, while there were records for only 14% of Riodinidae (Fig. 2).

The present compilation included 51,425 records, of which 44,593 have valid information on butterfly-host plant associations (valid butterfly names at species level and valid host plant names at family, genus or species level), and a further 226 records refer to non-plant resources (detritivore or insectivore). The remaining records (6,606) are incomplete, dubious or generic records. Among the valid records, 58% had complete taxonomic information of plants (at species level), while an additional 35% had information at genus level.

The valid records included 5,146 butterfly species from 1,193 genera, that corresponds to 29% of the butterfly species and 66% of the genera estimated to occur worldwide, according to this compilation (Table 1). In general, all subfamilies were well represented (above 60% of the genera reported worldwide), except Satyrinae, Heteropterinae and Pyrginae (54–55%) and the Riodinidae (40–46%, Table 1). Plant records include 6,008 host plant species, 2,289 genera and 212 plant families.

Butterfly species have been reported feeding on 204 angiosperm plant families that represent the most species rich plant families in the world (comprising about 94% of the species and 92% of the genera reported worldwide; [38]). However only 20% of these plant genera were actually recorded. In general, Fabaceae (by 1,007 butterfly species), and Poaceae (by 811 species) were the plant families most frequently used. At generic level, *Acacia* (by 155 spp.), *Poa* (by 125 spp.), *Citrus* (by 102 spp.), and *Quercus* (by 100 spp.) were the most frequently used host plant genera. At species level, the most frequently reported host plants were mostly widespread or cultivated plants such as *Oryza sativa* (by 56 spp.), *Saccharum officinarum* (by 52 spp.), *Poa annua* (by 44 spp.), *Cocos nucifera* (by 44 spp.), and *Medicago sativa* (by 42 spp.). Only 276 species have recorded associations with non-angiosperm plants, or non-plant resources.

### Phylogenetic Pattern in Host Plant Association

**Figure 3:**
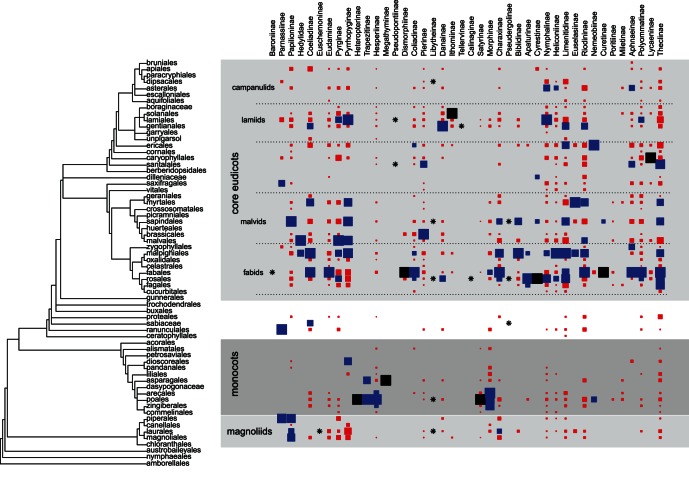
Graphical representation of the butterfly host plant association matrix. The squares represent the proportion of butterfly species in each subfamily that feed on a plant order (*z_ij_*). Only important resources are shown, colors denote values between 0.1< *zij* ≤0.5 (red), 0.5< *zij* ≤0.9 (blue), and *zij*>0.9 (black). The stars (*) denoted subfamilies with 15 or less species.

There was a notable disparity in host plant associations among butterfly subfamilies, even those that belong to the same family. Six butterfly families used magnoliids to some extent, but these plants only seem to be an important resource for three subfamilies: Papilioninae (on Piperales, Magnoliales and Laurales), Parnasiinae (Piperales), and Charaxinae (Laurales). The only species of Euschemoninae, as well as one of the five species of Lybiteinae, feed on Laurales (Fig. 3).

Six families used monocots, especially Poales, which is used by 891 butterfly species and is the second most used plant order overall. Poales was the primary resource for Satyrinae and Heteropterinae, the most important resource for Hesperiinae and Trapezitinae, and of some importance for Morphinae and few species in Lybiteinae and Nemeobiinae. The order Asparagales was the primary resource for Megathyminae, and was an important resource for Trapezitinae. Arecales was the most important host plant order for Morphinae, but was also of some importance for Hesperiinae. Zingiberales was important for Morphinae and Hesperiinae whereas Dioscoreales was important for Pyrrhopyginae. Records on basal eudicots were sparingly distributed, but Sabiaceae was important for Coeliadiinae and Pseudergoliinae, and Ranunculales was the most important order for Parnasiinae.

All seven families, and 36 of 41 subfamilies feed on rosids (fabids+malvids), including more than 90% of the records for Apaturinae, Baroninae, Biblidinae, Calinagynae, Curetinae, Dismorphiinae and Hedylidae. There were, however, two important gaps: the groups feeding on monocots, and the danaine clade (Danainae, Ithomiinae and Tellervinae) of Nymphalidae that fed on lamids (see below). Three of the four most frequently used orders were in the fabid clade: Fabales (by 1,009 spp.), Malpighiales (by 693 spp.) and Rosales (by 522 spp.). Fabales was the primary resource for Baroninae, Curetinae and Dismorphinae, and was the main resource for Coliadinae, Eudaminae, Polyomatinae, Charaxinae, Riodiniinae, and Theclinae. Plants of the Malpighiales were the main resource for Heliconiinae, Biblidinae, Coeliadinae, and Limenitidinae. Rosales was the primary resource for Calinaginae, Lybiteinae and Cyrestinae, and was the main resource for Apaturinae and Pseudergolinae.

Within the malvids, the orders Sapindales (420 spp.), Malvales (281 spp.), and Brassicales (204 spp.) were amongst the ten most used plant groups, but only a few butterfly subfamilies use them as the most important resource: Euselasiinae on Myrtales, Pierinae on Brassicales, Papilioninae on Sapindales, and Pyrginae and the family Hedylidae on Malvales.

Within basal asterids, the Santalales, Caryophyllales and Ericales were used by ca. 200 species each. Santalales was used by the only species of Pseudopontinae and was also important for the Pierinae and the Theclinae. Caryophyllales was the primary resource for Lycaeninae, while Ericales was the main resource for Nemeobiinae, and was also important for Limenitidinae and Coeliadinae.

Within Lamiids, Gentianales was used by 204 butterfly species, and Lamiales was used by 421 species. Gentianales was used by the only species of Tellervinae, was the main resource for Danaiinae, and was also important for Limenitidinae, Coeliadinae and Riodiniinae. Lamiales was used by the only species of Pseudopontinae and was the main resource for Nymphalinae and Pyrrhopyginae, but also important for Polyommatinae and Pyrginae. Solanales was the primary resource for Ithomiinae.

Many butterfly subfamilies have single records on Capanulids, but only the Asterales was important for Nymphalinae, Aphnaeinae, and Heliconinae, and the Dipsacales was used by one species of Lybitheinae.

### Relation between Host Plant Diversity and Butterfly Species Richness

All measures of host plant diversity were higher for intermediate to high values of butterfly species richness. Typically a subfamily with 500 or more species would use >25 host plant orders, but since many of these are either used by few species or are closely related, the values of α and PD are between six and nine (Table 2). Only Satyrinae, and to some extent Hesperiinae, showed lower host plant diversity with high species richness. However, for all subfamilies the observed values of PD were either similar or significant lower (p<0.05) than the value of PD expected from a random sample with a similar value of *h* (Table 2).

**Table 2:** Host shift transition rates (+/− S.E.) among plant orders and non-plant resources for the three possible butterfly phylogenies.

Alternative 1						
	Animal resources	Non angiosperm	magnoliids	monocots	basal eudicots	core eudicots
Animal resources				fixed at 0		
Non angiosperm						
magnoliids						
monocots		0.132+/−0.066		0.619+/−0.244		7.346+/−2.845
basal eudicots						
core eudicots						
Alternative 2						
Animal resources				fixed at 0		
Non angiosperm						
magnoliids						
monocots		0.137+/−0.068		0.617+/−0.244		7.301+/−2.868
basal eudicots						
core eudicots						
Alternative 3						
Animal resources				fixed at 0		
Non angiosperm						
magnoliids						
monocots		0.122+/−0.061		0.612+/−0.249		7.265+/−2.825
basal eudicots						
core eudicots						

In general there was a significant (p<0.001) and strong positive correlation between host plant diversity measures and the logarithm of butterfly species richness. Correlations, based on number of taxa (*h*), were lower than those based on phylogenetic information (PD) or the association matrix **C** (α). Similarly, using phylogenetic independent contrasts resulted in higher correlation, and these results were similar for alternative phylogenies (Table 3).

**Table 3:** Correlation between measures of host plant diversity with butterfly species richness.

				α			PDrand		
Family	Subfamily	Number of species	*H*	Mean	SE	PDobs	Mean	SD	p (PDobs ≠ PDrand)
Papilionidae	Baroniinae	1	1	0	–	1	–	–	–
	Parnassiinae	61	7	2.277	1.036	3.889	3.781	0.712	0.565
	Papilioninae	400	26	6.306	1.418	6.19	8.676	1.025	0.008
Hedylidae	Hedylidae	36	4	0.935	0.863	1.397	2.556	0.604	0.032
Hesperiidae	Coeliadinae	89	21	9.966	2.848	5.968	7.55	0.97	0.041
	Euschemoninae	1	1	0	–	1	–	–	–
	Eudaminae	430	26	7.325	1.731	6.54	8.595	1.054	0.025
	Pyrginae	642	26	6.708	1.562	6.365	8.645	1.024	0.011
	Pyrrhopyginae	490	20	6.426	1.726	4.952	7.327	0.967	0.007
	Heteropterinae	182	1	0.241	0.276	1	–	–	–
	Trapezitinae	75	2	0.409	0.324	1.079	1.347	0.569	0.194
	Hesperiinae	2,020	25	5.438	1.228	6.111	8.409	1.036	0.013
	Megathyminae	39	1	0.191	0.212	1	–	–	–
Pieridae	Pseudopontiinae	1	2	0	–	1.254	1.392	0.565	0.395
	Dismorphiinae	58	3	1.090	0.775	1.286	2.038	0.571	0.084
	Coliadinae	180	20	5.494	1.486	5.254	7.310	0.996	0.018
	Pierinae	761	29	7.62	1.642	6.571	9.207	1.04	0.004
Nymphalidae	Libytheinae	10	5	4.632	3.325	3.635	3.026	0.651	0.824
	Danainae	167	19	6.192	1.711	5.46	7.126	0.985	0.042
	Ithomiinae	339	7	1.774	0.774	2.889	3.803	0.676	0.085
	Tellervinae	7	1	0		1	–	–	–
	Calinaginae	10	1	0		1	–	–	–
	Satyrinae	2,292	12	2.034	0.679	4.111	5.357	0.866	0.068
	Morphinae	245	18	5.477	1.536	5.286	6.823	0.977	0.048
	Charaxinae	342	25	6.312	1.456	5.698	8.437	1.041	0.005
	Pseudergolinae	7	3	1.989	1.651	1.889	2.042	0.576	0.332
	Biblidinae	275	11	3.023	1.065	3.254	5.100	0.839	0.010
	Apaturinae	87	5	1.383	0.724	2.571	3.023	0.631	0.201
	Cyrestinae	46	4	1.594	1.001	2.381	2.572	0.595	0.300
	Nymphalinae	509	33	8.02	1.601	6.651	10.065	1.046	0.001
	Heliconiinae	562	29	7.088	1.51	7.143	9.222	1.078	0.032
	Limenitidinae	1,023	31	8.21	1.713	7.73	9.672	1.107	0.043
Riodinidae	Euselasiinae	171	5	2.212	1.273	2.873	3.055	0.627	0.39
	Riodininae	1,138	30	8.66	1.863	7.873	9.471	1.026	0.06
	Nemeobiinae	82	3	1.128	0.807	2.444	2.028	0.562	0.75
Lycaenidae	Curetinae	18	2	0.797	0.708	1.238	1.334	0.562	0.44
	Poritiinae	721	6	–	–	–	–	–	–
	Miletinae	188	7	–	–	–	–	–	–
	Aphnaeinae	286	19	5.897	1.615	4.460	7.129	0.998	0.005
	Polyommatinae	1,477	32	6.732	1.338	7.413	9.815	1.085	0.014
	Lycaeninae	110	8	2.328	0.971	2.762	4.164	0.747	0.022
	Theclinae	2,276	39	7.974	1.43	8.413	11.259	0.99	0.004

*h* = simple richness of host plant orders. α = Fishers’s alpha. PD = Faith’s index of Phylogenetic Diversity based on plant phylogeny, with values observed (obs) and expected under random sampling of the phylogeny (rand).

### Congruence Analysis

The global test for congruence for matrix **A** was not significant (*p* = 0.157), but 17% of the 570 links were apparently significant (*p*<0.05), as might be expected for systems with a mixed structure containing a partial coevolutionary structure with additional random shifts in hosts use. However, in this situation the tests of individual links have inflated type I error, and an adjusted significance level should be used to identify truly significant links [50]. With *p*<0.03 the number of significant links reduces to only three, suggesting that these relationships are almost completely spurious.

Fitting the model to the matrix of important links, **X** (more than 10% of the species in each subfamily, 113 links), resulted in a significant global test (*p* = 0.004). In this situation, the nominal significance level for the link-tests are valid [48], (*p*<0.05), and 56.6% of the associations were found to be significant according to the parameter ParaFit2.

**Figure 4:**
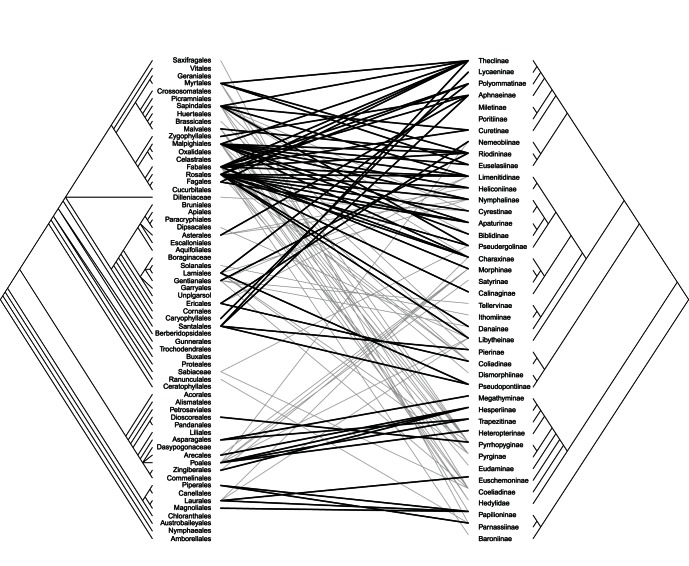
Congruence among plant (right) and butterfly (left) phylogenies. Lines between the phylogenies indicate associations based on the interaction matrix of important links (**X**), black lines represent congruent links (p<0.05) according to the ParaFitLink2 test. Based on the alternative 1 cladogram.

Congruent links were found between the Papilionidae-magnoliids, Hesperiidae-monocots (including Pyrrhopyginae-Dioscoreales), Pieridae with asterids, and Nymphalidae, Riodinidae and Lycaenidae with rosids and some asterids (Fig. 4). Interestingly, Baroninae, Hedylidae, and the basal Hesperiidae, and the danaine clade of Nymphalidae do not show significant congruent links.

Results with a traditional phylogeny were very similar (global test p = 0.132, 4% of significant links for matrix **A** and p = 0.002, 47.8% of significant links for matrix **X**), but with the alternative 2 phylogeny, both matrices were significantly congruent (global test p = 0.042 with 39.8% of significant links for matrix **A** and p = 0.003 with 42.5% of significant links for matrix **X**).

### Ancestral Character Estimation (ACE)

The simplified model was slightly favored by the AIC-criterion (AIC_simple_ = 140.6 *vs.* AIC_full_ = 142.6 and AIC_null_ = 148.9). In the selected model, the transition rate towards core-eudicots was the highest, with very low rates towards the basal groups (Table 4). The models for the other butterfly phylogenies were very similar in AIC support and rate estimates (Table 4) and resulted in similar estimates of ancestral character. We therefore only present the results for the first alternative.

**Table 4:** Pearson’s product moment correlation between logarithm of butterfly richness and three measures of host plant diversity using raw data and phylogenetic independent contrasts.

		Phylogenetic contrast		
	Normal correlation	Alternative 1	Alternative 2	Traditional
df	38	37	37	37
*h*	0.782	0.754	0.802	0.800
*α*	0.695	0.959	0.958	0.920
PD	0.792	0.979	0.979	0.980

df = degrees of freedom for the correlation test. *h* = simple richness of host plant orders. α = Fishers’s alpha. PD = Faith’s index of Phylogenetic Diversity based on plant phylogeny. All correlations were significant (p<0.05).

**Figure 5:**
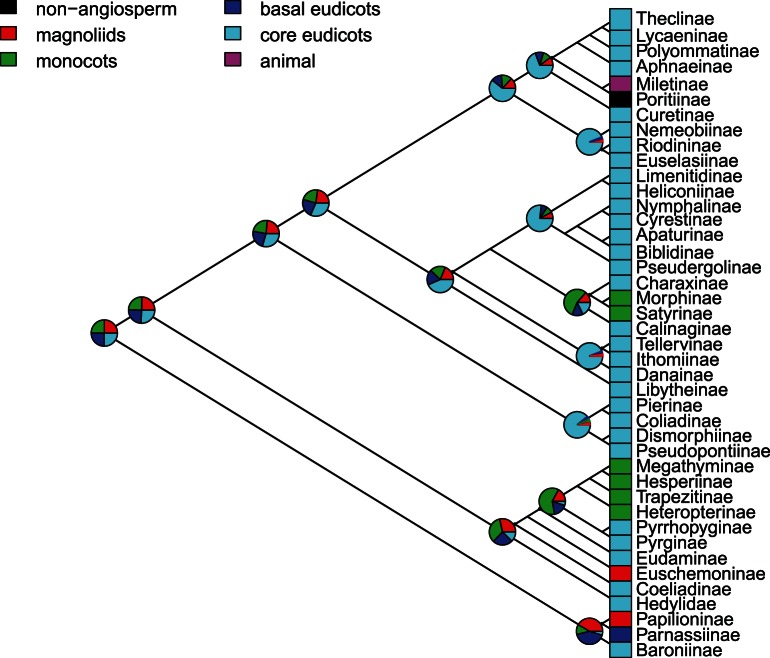
Likelihood of ancestral host plant in the butterfly phylogeny. Blocks on the right represent the observed states for each subfamily, piecharts represents the scaled likelihood of each potential ancestral character at selected nodes in the phylogeny. Based on the alternative 1 cladogram.

There was no conclusive evidence for a common ancestral state with the alternative 1 phylogeny (scaled likelihood around 0.25 for all four groups), but there seem to be at least three different lineages: 1) the most likely ancestral state for Papilionidae was equally likely to be the magnoliids or the basal eudicots (0.451); 2) Hesperiidae-Hedylidae were more likely to be originally associated with monocot- (0.445) or magnoliid-feeding (0.269), with a later shift to core-eudicots; 3) The ancestral character remained unresolved in the Nymphalidae, but with a slightly higher likelihood (0.295) of core-eudicots compared to the basal groups; 4) for all other groups the ancestral character estate was most likely within core-eudicots: 0.751 for Pieridae, 0.493 for Lycaenidae and 0.403 for Riodinidae (Fig. 5).

## Discussion

The present analysis provides a first step for a comprehensive and quantitative review of butterfly diversity and their associations with host plants at the level of plant orders and butterfly subfamilies. The pioneering work by Ehrlich and Raven [2], and the broad-scale phylogenetic analysis of Janz and Nylin [7] considered around 400–450 taxa (including a mixture of species and genera), while the present compilation includes almost three times as many butterfly genera, representative of all bioregions and all currently recognized subfamilies.

A key result from this effort was that, despite the frequently mentioned incompleteness of host plant information for tropical species, we were able to compile records for an important proportion of species in the three tropical regions analyzed (NT, OR and AT). Although NT was the region with the most incomplete dataset, it was also the region with the highest absolute numbers of species with host plant information (Table 1. Fig. 2). Gaps in knowledge are more striking precisely in species-rich taxa and regions, where rare species make up a large proportion of the species pool [55]. In these cases, the lack of field observations might lead to underestimates of host plant use, but even so the data are likely to be representative of larger patterns. For example, Satyrinae is one of the most speciose subfamilies among Nymphalidae, with 2,292 species known worldwide [25], [56], but despite its high diversity it has only been recorded on eleven plant orders (Fig. 3). The 414 species of Satyrinae compiled in this study represent one of the largest absolute values for any subfamily, which provides a good representation of the taxonomic diversity of this group (49% of the known genera), even though they result in a low proportion of the subfamily total (18%; Table 1). Fieldwork in tropical areas like the ACG in Costa Rica confirms the predictions of previous authors that most rare Satyrinae would turn out to feed on grasses [2], [14].

Clearly the completeness of the present database was only possible thanks to the availability of digital resources, which represent an important opportunity for the analysis of biotic associations [57]. Host plant-associations and distribution records, tools for validation of taxonomic and nomenclatural information, and detailed phylogenies for both taxonomic groups, were all available in different sources thanks to the contribution of several individuals and research groups. However, validating large amounts of isolated data and keeping this information up to date represent major challenges for online services [58]. The heterogeneity in the quality of data compiled required careful revision and checking in order to combine them into a useful quantitative dataset. Nevertheless, the results are useful for evaluating the role of host plant diversity in butterfly diversification and for addressing questions regarding the macroevolutionary patterns in host plant association.

### Correlation between Host Diversity and Butterfly Species Richness

If herbivore species richness has been promoted by the diversification of the plants they interact with, there should be a general correlation between host plant diversity and butterfly species richness [17]. Indeed, a significant and strong correlation between host plant diversity and butterfly species richness was found, and this was even higher when phylogenetic relationships among butterflies was considered (Table 3). Characteristic examples of this correlation are evident in the Theclinae, Nymphalinae and Pierinae (Table 2). Hesperiinae and Satyrinae are important outliers in this general trend: both had extraordinary species richness (represent 56.5% of all hesperiids, and 42.6% of all nymphalids respectively), combined with very low host plant diversity that was mainly restricted to monocots. The importance of Hesperiinae and Satyrinae has been clearly understated in most discussions on butterfly diversification and host plant diversity (in fact, Janz et al. [17] reduced Satyrinae to a single clade in their analysis), and deserves more attention in the future. Even considering these two important outliers, the correlation between butterfly species richness and host plant diversity seems to be more robust than initially believed [17].

Host plant diversity can be both a cause and a consequence of butterfly species diversification [8], and this association should be analyzed in a phylogenetic and historical context in order to quantify the relative contribution of biotic interactions [59], climate change [41] and biogeographical history [50]. We will attempt to evaluate two macroevolutionary questions with the compiled information: whether evolution of host plant use has followed consistent patterns across butterfly lineages, and if there is a common ancestral host for all butterfly lineages.

### Macroevolutionary Patterns in Host Plant Association

Our results suggest that, under the current view of butterfly phylogeny, there are significant congruencies with the phylogenies of plant orders. We were able to identify three main groups of congruent links: (1) Papilionidae with magnoliids, (2) Hesperidae with monocots, and (3) Pieridae, Lycaenidae, Riodinidae and Nymphalidae with the eudicots, particularly fabids and malvids, and few asterids (Fig. 4). These were also recovered as the most probable ancestral states (Fig. 5). As other authors have previously pointed out, a strict congruence does not necessarily mean that a continual association has occurred between two clades [3], [5]. This at least requires that the two clades be of similar age [3]. The relative timing of adaptive radiations in host plants and butterfly is controversial. Although the major angiosperm radiation occurred **∼**140 to 100 million years ago (Mya), and fossil data suggest that angiosperm feeding Lepidoptera were already present ∼97 Mya, butterflies probably radiated long after their host plants (**∼**75 Mya) [26], [60], [61]. This hypothesis of recent butterfly origin necessarily implies a very limited role, if any, for stepwise coevolution in butterfly diversification [62], [63]. However, others posit a much older age of butterflies (**∼**100 Mya), with speciation influenced by angiosperm evolution and the breakup of the supercontinent Gondwana [50], [64], [65].

Beside the incongruences in timing of diversification between host plants and butterflies, the high frequency of apparently random host plant shifts – represented by a large number of marginal associations (<10% of the species in each subfamily), and >40% of non-significant links in the Parafit analysis – also points to a more complex scenario of ancestral relationships and makes the interpretation of congruence patterns more difficult. Nylin and Wahlberg [66] suggested that some shifts are more probable, either because of an easier return to the ancestral state, or because a group of hosts is more favorable. Our results from ACE models showed a large difference in the transition rates from the other angiosperms toward the eudicots, with only one major shift from eudicots to monocots (Table 4). This result agrees with those reported by Janz and Nylin [7] and provides support for the oscillation hypothesis as an alternative explanation for butterfly diversification.

Alternative topologies had large effects on estimates of congruence, but not on the estimation of ancestral characters. Analyses based on modern butterfly phylogeny (alternative 2 cladogram), suggest more significant congruencies, with 39–42% of significant links. Clearly a deeper knowledge of butterfly family-level relationships is necessary to resolve these discrepancies and highlights the importance of developing comprehensive phylogenetic studies combining molecular and morphological data [26], [39], [67].

Our approach to reconstruct ancestral states is based on the most commonly used resource for each subfamily. This may not be the original host if, for example, a clade of butterflies has colonized and radiated on an apomorphic resource. In fact, the basal groups within the Papilionidae and the Hesperiidae-Hedylidae clades show different associations from the most diverse clades (Figs. 4 and 5) and this can lead to different interpretations (see below). Future analysis should combine this dataset with genus- and species-level butterfly phylogenies to shed more light on this issue.

### The Larger Picture

Our study contributes to the visualization of the complex pattern of interactions at family level and provides a context to discuss the potential mechanisms that might explain the macroevolutionary pattern of host plant association observed at lower levels. Detailed studies at family or subfamily levels highlight the role of host plant association in the diversification of specific groups, and reveal the importance of the timing of host shifts and changes in paleoclimate and paleohabitat.

The most likely ancestral host of Papilionidae is in the Aristolochiaceae (order Piperales within the magnoliids, Fig. 5) [68], although the basal position of the Baroniinae has been used as an argument to suggest fabid-feeding as the original state for this family [2], [7], [10]. This family shows a prominent latitudinal gradient in species richness and host plant specialization [69], but a detailed phylogenetically integrated approach has shown that diversification of tropical species was more related to climate than to host plant association, whereas both factors seem to affect diversification in temperate clades [68].

The biggest discrepancy between our analysis and previous results is about the ancestral host of the Hedylidae/Hesperiidae clade. The relationship between Hedylidae and Hesperiidae has only been pointed out in a recent analysis of the redefined Papilionoideae [26], but the associations of Hedylidae and basal Hesperiidae were already used as an argument in favor of malvales as an ancestral host plant for all butterflies [10]. However, we found that feeding on monocots is a more likely ancestral state (Fig. 5). The host plant relationships of Hesperiidae were included as characters in a phylogenetic analysis of the group by Warren et al. [67] and the resulting phylogeny implied a single major switch from dicot to monocot feeding among the Hesperiidae (presumably by the ancestor of Heteropterinae, Trapezitinae and Hesperiinae). The host switch was accompanied by considerable diversification, especially in the New World Moncini and Hesperiini. Under this scenario, there have been just a few secondary gains of monocot feeding among dicot-feeding lineages, and only a few reversals back to dicot feeding among monocot-feeding lineages [67]. However the authors only distinguished between monocot and dicot (eudicot+magnolids) feeding and did not include complete and quantitative data on host plant associations to test this assumption explicitly. Our observations suggest that host range in Hesperiidae is very diverse, including 44 orders across the whole plant phylogeny (Fig. 3), and thus the estimation of the ancestral state is more difficult (Fig. 5). A more detailed assessment of the associations within this clade is needed, especially to account for the scattered records of basal Hesperiidae in the magnoliids and monocots, including the only species of Euschemoniinae on Laurales and several records of Pyrrhopyginae on magnoliids and dioscoreales (Fig. 3).

In the remaining components of the butterfly phylogeny, the core-eudicots dominate as host plants and most likely represent the ancestral host for each group, with only one major shift toward monocots and a few particular shifts to other hosts (Figs. 4 and 5) [13], [59], [66], [68]. A series of host-shifts within the Pieridae appears to be linked to extraordinary radiation of the subfamily Pierinae [40] and involve an initial diversification on Brassicaceae, followed by a second and probably larger diversification on parasitic plants in the order Santalales (basal asterids), and later colonization of the hosts of these parasitic plants. The host plant associations of many Pierinae remain unknown, but it seems that the larger genera *Delias, Catasticta* and *Mylothris* are mostly restricted to Santalales [42], [65], [70]. However, diversification in these large genera is probably only partially related to host plant use [71] and much more due to geographical isolation in tropical mountains during periods of climatic change [40].

The Nymphalidae include several families with both low and high diversity of species and restricted or generalized host plant associations [12], [17], [26], [72]. The subfamily Nymphalinae shows an elevated diversity in host plant use, which could be caused by ancestral polyphagy [73], and it has been proposed that the evolutionary trend is actually towards increased generalization rather than specialization [17]. In contrast, the diversification of Satyrinae seems to have followed a shift to feeding on monocots and may be linked to the radiation and expansion of Poales as a dominant plant form after climatic changes created suitable new habitats for colonization by grasses [50]. Current estimates of the tentative time frames of these events confirm this is a plausible sequence (origin of Poales, radiation of Poales, origin and diversification of Satyrinae), and could explain the diversification of some of the most complex Satyrinae groups (tribes, subtribes and genus-groups) [41].

Finally, within the Lycaenidae the extreme diversification in the Theclinae has been previously linked to their strong associations with ants, which might also be partly responsible for frequent host shifts [1], [74]. This in turn could explain the higher host plant diversity for Theclinae that was found in this study and previous studies [14], [74]–[76], and may also explain the species diversity in other subfamilies in the Lycaenidae and Riodinidae [77]. Recently, Megens et al. [78] suggested that the timing of a basal radiation in *Arhopala* (the most speciose genus of Theclinae, with 9% of the species in Southeast Asia) coincided with major climate changes commencing during the middle Miocene. These climatic changes could have produced massive floristic changes in the rainforest of the Southeast Asian tropics, dominated by trees of the family Dipterocarpaceae. Preadapted *Arhopala* species may have been able to fully exploit the newly formed dipterocarp rain forest emerging some 10–15 Mya, resulting in massive speciation in this genus of butterflies.

## Conclusion

The data compiled here represent host records for nearly one third of all butterfly species (∼29%) and 58% of these records had complete taxonomic information on host plants (at species level). Despite limitations in the dataset, it is an important step towards assembling and analysing standardized information about host plant association for this important group of insects. As such, it can be used to evaluate macroecological hypotheses such as tests of latitudinal gradients in species richness and patterns of host specialization (monophagy vs polyphagy). Here we give the first quantitative account of host plant associations for all seven butterfly families at a global scale and describe macroevolutionary patterns in host plant associations.

We found a positive correlation between host plant diversity and butterfly diversification and a congruent association between the phylogenies of plants and butterflies. However, we also detected a high number of random associations that could be interpreted as host shifts that might have helped to promote the diversification of certain butterfly lineages [8]. The congruent associations are also within the most likely ancestral hosts of each butterfly clade and tend to show a large agreement with previous analyses [13], [59], [66], [68]. The one exception is Hesperiidae where the ancestral host seems to be within the monocots and not the dicots [18]. These results should be combined with studies of selected clades to assess the relative importance of changes in host plant associations through evolutionary time.

## Supporting Information

Dataset S1 **Compressed R-data file with objects used in the analysis.** The file contains the association matrices (Aij, Cij Zij and Xij), the butterflies phylogenies (Alternative1.tree, Alternative2.tree and Traditional.tree), plant phylogeny (APGorders.tree), and summary table (Summary.table).
(CSV)

Dataset S2 **File in comma separated value format used to build** **Figure 2** **.** The file contains the number of butterfly species and number of butterfly species with host plant records among regions and subfamilies.
(CSV)

Text S1 **Text file with example of R-code.** The file contains commented R-code to use with the Dataset S1.
(PDF)
