# Supplementary material for: Congruence and Diversity of Butterfly-Host Plant Associations at Higher Taxonomic Levels
Source: PLoS One. 2013 May 23;8(5):e63570. doi: 10.1371/journal.pone.0063570 (PMC3662771; doi:10.1371/journal.pone.0063570)
Supplement: Text S1 — Text file with example of R-code. The file contains commented R-code to use with the Dataset S1. (PDF) [file pone.0063570.s003.pdf]

Supplementary Text for the Article *Congruence and diversity of butterfly-host plant associations at higher taxonomical levels*

JR Ferrer-Paris, AY Sánchez-Mercado, AL Viloria, and J Donaldson
Centro de Estudios Botánicos y Agroforestales and Centro de Ecología – Instituto Venezolano de Investigaciones Científicas
Applied Biodiversity Research Division – South African National Biodiversity Institute
Botany Department – University of Cape Town

Version of March 22, 2013

The Dataset S1 is provided in order to allow users to re-analyse the data using alternative classifications, including the transfer of genera to different subfamilies as the ones considered in the article. Please note that updates to the compilation and data sets will be posted in `http://papilionoidea.myspecies.info`.

All analyses were performed using *R* (R Development Core Team, 2010), but the data can be easily exported for use with other software. Within *R* we used the *ape* package (Paradis *et al.*, 2004):

```
> library(ape)
```

Substitute `data.dir` with the working directory in your system, then load the data:

```
> data.dir <- "~/CEBA/Rdata/"
> load(paste(data.dir,"DatasetS1.rda",sep=""))
```

You can plot the phylograms with the functions in package `ape`:

```
> plot(Alternative1.tree,
+      tip.color=c(rep("orangered",3),"pink",rep("brown",9),
+        rep("green",4),rep("orange",15),rep("cyan",3),
+        rep("slateblue",7)),cex=.7)
```

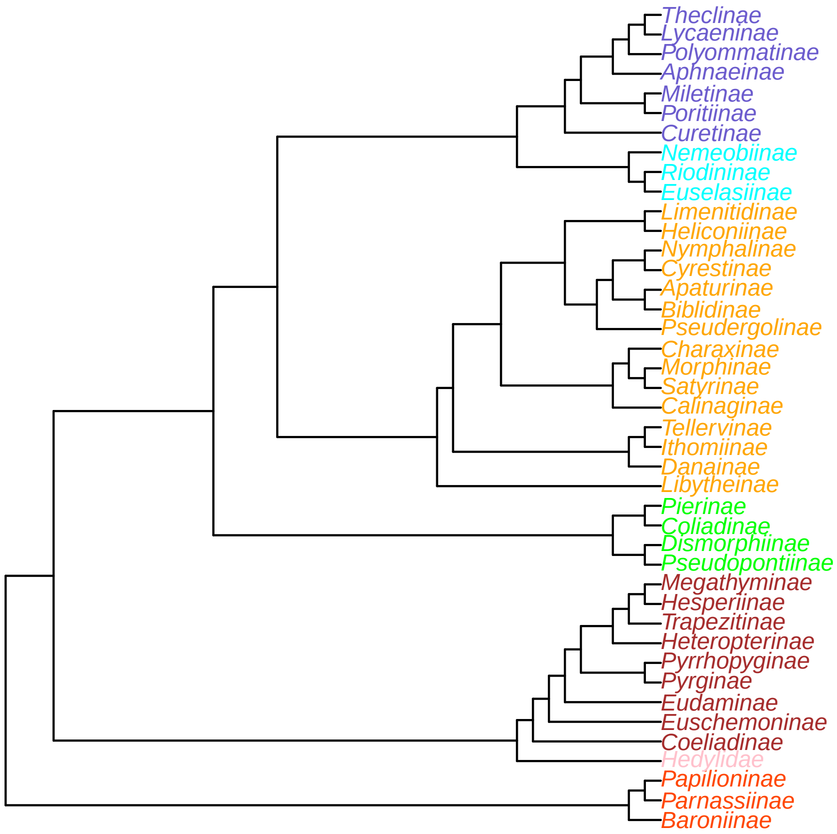

The object `Summary.table` shows how many species of each genus feed on a particular plant order, and how the genera were assigned to each subfamily in our compilation.

```
> dim(Summary.table)

[1] 1193   63

> Summary.table[1:6,1:6]

          Genus  Subfamily Alismatales Apiales Aquifoliales Arecales
76      Apatura Apaturinae           0       0            0        0
119 Asterocampa Apaturinae           0       0            0        0
```

```
248    Chitoria Apaturinae             0       0             0        0
333      Dilipa Apaturinae             0       0             0        0
348    Doxocopa Apaturinae             0       0             0        0
409   Eulaceura Apaturinae             0       0             0        0
```

If necessary, the assignment of genera to subfamily can be changed, or the total number of species feeding in a particular host plant order can be updated. Then the R-function `aggregate` can be used to provided totals for each subfamily:

```
> new.C <- aggregate(Summary.table[,-(1:2)],
+                    by=list(Subfamily=Summary.table$Subfamily),
+                    sum)
> new.C[1:6,1:7]
```

```
    Subfamily Alismatales Apiales Aquifoliales Arecales Asparagales Asterales
1  Apaturinae           0       0            0        0           0         0
2  Aphnaeinae           0       1            0        0           0        14
3  Baroniinae           0       0            0        0           0         0
4  Biblidinae           0       0            0        0           0         0
5 Calinaginae           0       0            0        0           0         0
6  Charaxinae           0       2            0        0           1         1
```

The resulting matrix is equivalent to the association matrix **C** in the article.

The original matrix used in the analysis are stored in R-objects `Aij`, `Cij`, `Xij` and `Zij`. The following code can be used to make a graphic representation of matrix A.

```
> par(mar=c(1,3,4,1))
> layout(matrix(c(1,2,2),ncol=3))
> plot(APGorders.tree)
> par(xpd=F)
> plot(1,1,xlim=c(1,ncol(Aij)),ylim=c(1,nrow(Aij)), type="n",axes=F,xlab="",ylab=""
> par(xpd=NA)
> text((1:ncol(Aij))-.4,rep(65,ncol(Aij)),colnames(Aij),srt=90,cex=.7,pos=4)
> par(xpd=F)
> for (j in 1:ncol(Aij)) {
+         if (sum(Aij[,j])>0) {
+                 x <- rep(j,nrow(Aij))
+                 y <- 1:nrow(Aij)
+                 z <- Aij[,j]
+                 points(x[z>0], y[z>0], pch=8)
+         }
+ }
```

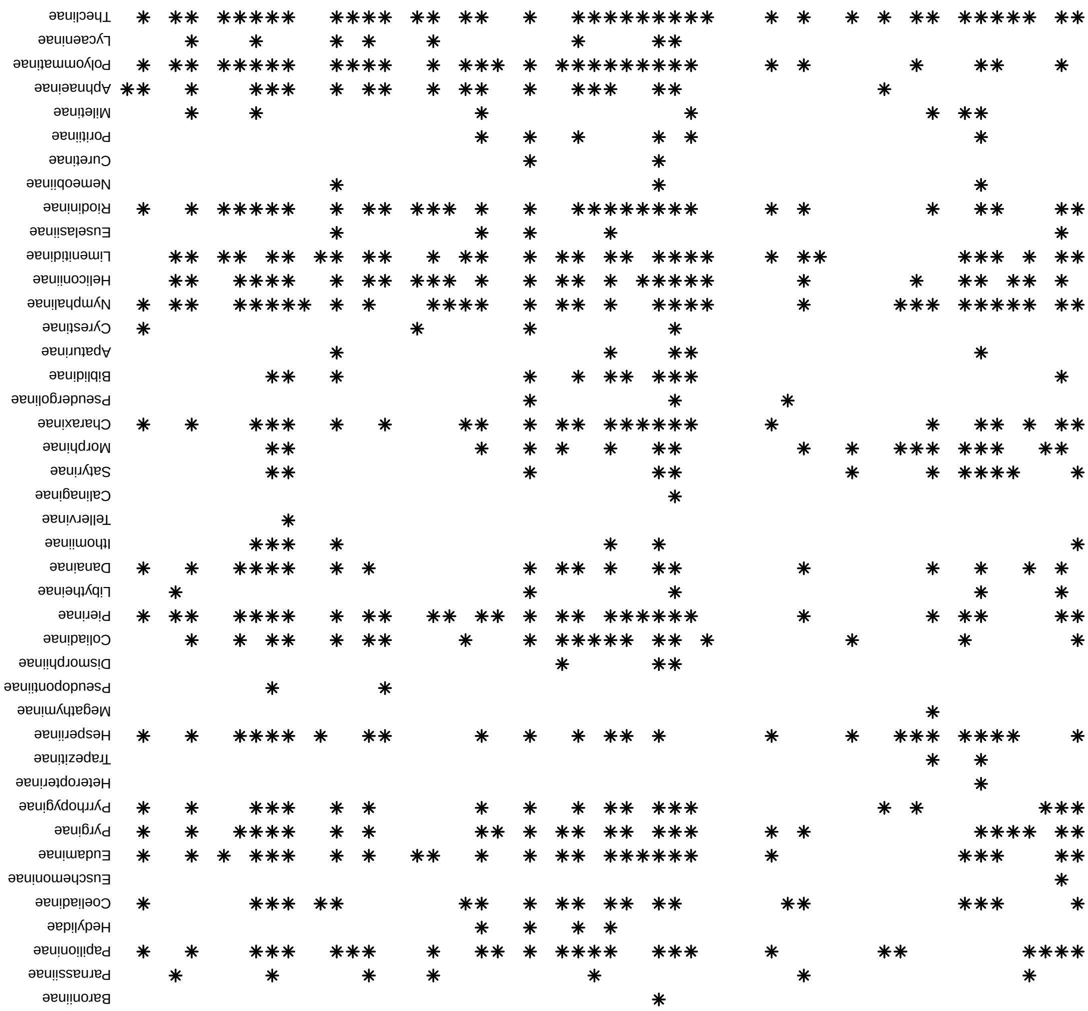
Theclinae
Lycaeninae
Polyommatinae
Aphnaeinae
Miletinae
Poritiinae
Curetinae
Nemeobiinae
Riodininae
Euselasiinae
Limenitidinae
Heliconiinae
Nymphalinae
Cyrestinae
Apaturinae
Biblidinae
Pseudergolinae
Charaxinae
Morphinae
Satyrinae
Calinaginae
Tellervinae
Ithomiinae
Danainae
Libytheinae
Pierinae
Coliadinae
Dismorphiinae
Pseudopontiinae
Megathyminae
Hesperiinae
Trapezitinae
Heteropterinae
Pyrrhopyginae
Pyrginae
Eudaminae
Euschemoninae
Coeliadinae
Hedylidae
Papilioninae
Parnassiinae
Baroniinae

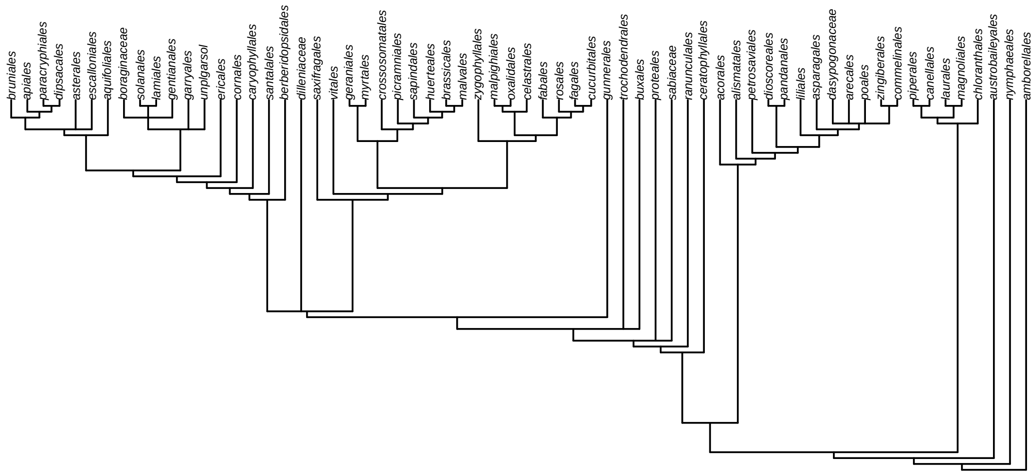
bruniales
apiales
paracryphiales
dipsacales
asterales
escalloniales
aquifoliales
boraginaceae
solanales
lamiales
gentianales
garryales
unplgarsol
ericales
cornales
caryophyllales
santalales
berberidopsidales
dilleniaceae
saxifragales
vitales
geraniales
myrtales
crossosomatales
picramniales
sapindales
huerteales
brassicales
malvales
zygophyllales
malpighiales
oxalidales
celastrales
fabales
rosales
fagales
cucurbitales
gunnerales
trochodendrales
buxales
proteales
sabiaceae
ranunculales
ceratophyllales
acorales
alismatales
petrosaviales
dioscoreales
pandanales
liliales
asparagales
dasypogonaceae
arecales
poales
zingiberales
commelinales
piperales
canellales
laurales
magnoliales
chloranthales
austrobaileyales
nymphaeales
amborellales

References

Paradis, E., Claude, J. & Strimmer, K., 2004. *APE: analyses of phylogenetics and evolution in R language*. Bioinformatics, 20:289–290.

R Development Core Team, 2010. *R: A Language and Environment for Statistical Computing*. R Foundation for Statistical Computing, Vienna, Austria. URL `http://www.R-project.org/`. ISBN 3-900051-07-0.
